# A longitudinal comparison of emotional, behavioral and attention problems in autistic and typically developing children

**DOI:** 10.1017/S0033291723001599

**Published:** 2023-12

**Authors:** N. Wright, V. Courchesne, A. Pickles, R. Bedford, E. Duku, C. M. Kerns, T. Bennett, S. Georgiades, J. Hill, A. Richard, H. Sharp, I. M. Smith, T. Vaillancourt, A. Zaidman-Zait, L. Zwaigenbaum, P. Szatmari, M. Elsabbagh, Pathways Team

**Affiliations:** 1 Department of Psychology, Manchester Metropolitan University, Manchester, UK; 2 Centre for Addiction and Mental Health, University of Toronto, Toronto, Canada; 3 Department of Biostatistics & Health Informatics, King's College London, London, UK; 4 Department of Psychology, University of Bath, Bath, UK; 5 McMaster University, Hamilton, Canada; 6 Department of Psychology, University of British Columbia, Vancouver, Canada; 7 School of Psychology & Clinical Language Sciences, University of Reading, Reading, UK; 8 IWK Health Centre, Autism Research Centre, Halifax, Canada; 9 Department of Primary Care and Mental Health, Faculty of Health and Life Sciences, University of Liverpool, Liverpool, UK; 10 Dalhousie University and IWK Health, Halifax, Canada; 11 University of Ottawa, Ottawa, Canada; 12 Tel Aviv University, Tel Aviv, Israel; 13 University of Alberta, Alberta, Canada; 14 Department of Neurology and Neurosurgery, McGill University, Montreal, Canada

**Keywords:** attention problems, Autism, behavioral problems, emotional problems, longitudinal, trajectories

## Abstract

**Background:**

Mental health problems are elevated in autistic individuals but there is limited evidence on the developmental course of problems across childhood. We compare the level and growth of anxious-depressed, behavioral and attention problems in an autistic and typically developing (TD) cohort.

**Methods:**

Latent growth curve models were applied to repeated parent-report Child Behavior Checklist data from age 2–10 years in an inception cohort of autistic children (Pathways, *N* = 397; 84% boys) and a general population TD cohort (Wirral Child Health and Development Study; WCHADS; *N* = 884, 49% boys). Percentile plots were generated to quantify the differences between autistic and TD children.

**Results:**

Autistic children showed elevated levels of mental health problems, but this was substantially reduced by accounting for IQ and sex differences between the autistic and TD samples. There was small differences in growth patterns; anxious-depressed problems were particularly elevated at preschool and attention problems at late childhood. Higher family income predicted lower base-level on all three dimensions, but steeper increase of anxious-depressed problems. Higher IQ predicted lower level of attention problems and faster decline over childhood. Female sex predicted higher level of anxious-depressed and faster decline in behavioral problems. Social-affect autism symptom severity predicted elevated level of attention problems. Autistic girls' problems were particularly elevated relative to their same-sex non-autistic peers.

**Conclusions:**

Autistic children, and especially girls, show elevated mental health problems compared to TD children and there are some differences in predictors. Assessment of mental health should be integrated into clinical practice for autistic children.

Autism is a lifelong condition characterized by high rates of co-occurring mental health problems. Three of the most frequent comorbidities are attention deficit and hyperactivity disorder (ADHD), anxiety disorders, and oppositional defiant disorder (Salazar et al., 2015; Simonoff et al., 2008). The majority of evidence on mental health comorbidities comes from mixed-age samples and there are few longitudinal accounts of the developmental course comorbidities, fewer still which compare to typical development (TD) and none of the comparison studies have spanned childhood starting at toddler age. This is important because we do not know whether the developmental trajectories observed in typically developing (TD) children (for example, elevated oppositional problems at toddler/preschool age which normatively decrease throughout childhood) are the same in autistic children.

Studies using longitudinal growth curve analysis in TD children from early childhood have shown that behavioral and attention problems (collectively referred to as externalizing) are elevated at toddlerhood/preschool age and decrease over childhood. The results from growth curve modeling studies of emotional (or internalizing) are less consistent, but most studies report an overall increase from toddler/preschool age onward (Fanti & Henrich, 2010; Gilliom & Shaw, 2004; Marçal, 2020). However, two studies of autistic children within general population cohorts have shown an increasing trajectory on behavioral, attention and emotional problems combined from age 3 to 7 years (Midouhas, Yogaratnam, Flouri, & Charman, 2013) and an increase for all three dimensions examined individually from age 4 to 7 years, followed by a decrease to age 13 (Colvert et al., 2021). In these studies, TD children showed a decreasing combined trajectory (Midouhas et al., 2013) and no change in behavioral and hyperactivity problems, but the same pattern to the autistic children of increasing emotional problems to age 7 followed by decreasing to age 12 (Colvert et al., 2021). These findings suggest that autistic children may not follow the same early trajectories of mental health problems as seen in TD. Studies using growth mixture modeling have shown that a subgroup of TD children show persistent trajectories of oppositional behavior from toddler age onward (Côté, Vaillancourt, LeBlanc, Nagin, & Tremblay, 2006; Kjeldsen et al., 2016) and it may be that relatively more autistic children follow this persistent elevated trajectory. However, the expected decrease in behavioral and attention problems in TD was not observed in these studies.

It is likely that children who receive an autism diagnosis and are retained in a general population questionnaire-based study are less representative of the broader autistic population than those recruited into studies designed to assess autistic children. In a sample of autistic children recruited at diagnosis (*Pathways in ASD*, the same sample analyzed in this report; hereafter *Pathways*), results using the Child Behavior Checklist (CBCL; Achenbach and Rescorla, 2000) showed a decreasing slope of externalizing problems from ages 2.5 to 5.5, which is consistent with TD. Emotional problems also decreased (Vaillancourt et al., 2017). The CBCL, commonly used in TD longitudinal studies, has separate forms with developmentally appropriate items to assess pre- and school-age problems. In comparison to the Strengths and Difficulties Questionnaire (SDQ; Goodman, 1997), the short screening instrument used by the two population studies cited above (Colvert et al., 2021; Midouhas et al., 2013), the longer CBCL measure may be more sensitive to developmental change.

Studies of adolescent mental health trajectories in autism have also identified more similar trajectories to TD when using longer measures. Emotional problems were found to increase in two studies using the CBCL and the Aberrant Behavior Checklist (Aman, Singh, Stewart, & Field, 1985) another lengthier measure compared to the SDQ, but decreased in one study that used the SDQ. Behavioral and attention problems decreased on all measures (Anderson, Maye, & Lord, 2011; Gotham, Brunwasser, & Lord, 2015; Stringer et al., 2020). Two publications have examined mental health trajectories over adolescence within the general population Avon Longitudinal Study of Parents and Children, using a lengthier self-report depression scale (Pender, Fearon, Heron, & Mandy, 2020; Rai et al., 2018) and the SDQ (Pender, Fearon, Heron, & Mandy, 2020). Rai et al. (2018) compared children with and without an autism diagnosis from age 9 to 16 and found divergence in self-reported depression trajectories in early adolescence but similar growth in late adolescence. On the same sample Pender, Fearon, Heron, & Mandy (2020) generated trajectory groups of autistic and mental health symptoms from age 9 to 16 years. The shape of the mental health trajectories was similar in the groups of adolescents with high, low, increasing and decreasing autistic symptoms, with greater divergence in shape in late adolescence. There were striking differences in level of mental health symptoms between the low and high autistic symptom classes. In sum, the existing longitudinal data on whether co-occurring mental health problems in autistic children follow a similar trajectory to TD are inconsistent. To date, no study has spanned childhood, beginning from toddler age when trajectories of persistent emotional and behavioral problems emerge (Cote, Vaillancourt, LeBlanc, Nagin, & Tremblay, 2006) up to late childhood. As adolescence begins at age 10 (World Health Organization, 2001) we define late childhood as age 9–10 years.

Predictors of mental health problems and their developmental course in TD have also been tested in autistic samples, as well as testing autism-specific variables. Similar to the TD literature, lower family income and maternal education have been associated with elevated mental health problems in autistic youth, with some differences in patterns of association with level *v.* growth and across different mental health dimensions (Gotham et al., 2015; Midouhas et al., 2013; Stringer et al., 2020; Vaillancourt et al., 2017). Lower IQ is associated with elevated levels of internalizing and externalizing problems in TD samples (Papachristou & Flouri, 2020). In autistic youth, however, higher IQ or language abilities have been associated with elevated emotional problems (Gotham et al., 2015; Salazar et al., 2015; Vasa, Keefer, McDonald, Hunsche, & Kerns, 2020). The two-population derived autistic samples did not show an association between low IQ and behavioral or attention problems (Salazar et al., 2015; Simonoff et al., 2008), but low language abilities have been associated with a less steep decline in behavioral and attention problems from childhood to adulthood (Stringer et al., 2020). Given the higher rates of mental health problems in autistic individuals compared to non-autistic individuals, it seems reasonable to predict that the level of autistic symptoms would be associated with a greater degree of mental health problems. However, findings have been inconsistent, with some studies finding no association with level (Anderson et al., 2011; Simonoff et al., 2008) or growth (Stringer et al., 2020) but other showing a positive association with level of oppositional and some specific anxiety disorder symptoms (Baribeau et al., 2020; Salazar et al., 2015).

In TD, there are clear sex-specific effects on mental health trajectories; boys show higher levels of behavioral and attention problems than girls, but these problems decrease in both boys and girls across childhood (Fanti & Henrich, 2010). Girls clearly show elevated emotional problems in adolescence, with inconsistent evidence for a sex difference in preschool or childhood (Carter et al., 2010; Mesman, Bongers, & Koot, 2001). In autism, some studies support elevated behavioral and attentional problems in boys (Mandy et al., 2012; Salazar et al., 2015) but many find no sex difference in level (Gadow, DeVincent, Pomeroy, & Azizian, 2004; Prosperi et al., 2021) or growth (Anderson et al., 2011; Stringer et al., 2020). For emotional problems, whilst some studies have reported elevated level (Mandy et al., 2012) or growth (Gotham et al., 2015) in girls most report no sex difference (Anderson et al., 2011; Gadow et al., 2004; McCauley, Elias, & Lord, 2020; Salazar et al., 2015; Stringer et al., 2020) and some an elevated level in males (Prosperi et al., 2021). Studies that have taken the approach of comparing autistic individuals to their same-sex, non-autistic peers have shown that girls are significantly more elevated on anxiety, depression, hyperactivity and behavioral symptoms compared to their same-sex non-autistic peers (Lundström et al., 2019; Rødgaard, Jensen, Miskowiak, & Mottron, 2021).^.^

In the present study we use data from two longitudinal cohorts with repeated assessment of anxious-depressed, behavior problems and attention problems to compare level and growth in mental health problems in autistic and TD children. We extend previous literature by examining growth patterns from toddlerhood to late childhood using the CBCL pre-school and school age forms. We use growth curve models to compare growth patterns and generate percentiles from the scores in the general population sample to quantify the potential differences between autistic and TD children. We addressed the following questions: (1) are autistic children consistently at elevated risk of mental health problems throughout childhood compared to TD?; (2) does the pattern of developmental change in mental health problems in autistic children differ from TD; (3) do the risk factors documented in TD (child sex, low family income and IQ) also predict mental health outcomes for autistic children; (4) is autism symptom severity associated with increased co-occurring mental health problems? This study takes advantage of common measurement and age-span of repeated assessments between two longitudinal child development studies: Pathways, a Canadian autism inception cohort, and Wirral Child Health and Development Study (WCHADS), a UK population cohort of TD children.

## Sample

### Autistic participants

*Pathways* in ASD is a longitudinal multisite cohort study of Canadian children recruited at the point of diagnosis (*n* = 421). All children aged between 2 years to 4:11 years and without any exclusion criteria who received an autism diagnosis from a clinician using the DSM-IV-TR (APA, 2000) criteria and confirmed by the Autism Diagnostic Observation Schedule (ADOS; Lord et al., 2000) and the Autism Diagnostic Interview-Revised (ADI-R Le Couteur, Lord, & Rutter, 2003) were eligible to participate in the study. Exclusion criteria included cerebral palsy or other neuromotor disorders, genetic or chromosomal abnormality, severe vision or hearing impairment or if they had a sibling already participating in the study. Following the initial assessment, conducted at age of diagnosis (*N* = 397 with CBCL data; mean age 3.41 years, s.d. = 0.76, 334 M; 63F), children were reassessed six months (*N* = 327; mean age 3.99 years, s.d. = 0.79; 283 M; 44F) and one year post-diagnosis (*N* = 301; mean age 4.51 years, s.d. = 0.76; 259 M; 42F) and then yearly from age 6 (*N* = 250, mean age 6.60 years, s.d. = 0.32, 216 M; 34F; *N* = 197, mean age 7.74 s.d. = 0.26, 259 M 42F; *N* = 210 mean age 8.73, s.d. = 0.20, 177 M; 33F; *N* = 158, mean age 9.71 years, s.d. = 0.22; 129 M; 29F, *N* = 175, mean age 10.76, s.d. = 0.24, 146 M; 26F). Parent-report questionnaires were given to families to complete at home and return, so the numbers providing CBCL data is lower than those providing any data at each time. The Pathways study is conducted in five sites across Canada: Halifax, Montreal, Hamilton, Edmonton and Vancouver. Data from all participants with at least a parent-report CBCL available at time 1 (*N* = 397, 334 M; 63F) were used in the subsequent analysis. The site distribution of these participants can be found in online Supplementary Table S2, participants' diagnostic information is described in online Supplementary Table S3 and S4, and sociodemographic characteristics in online Supplementary Table S5.

### TD participants

The WCHADS is a prospective epidemiological cohort study starting in pregnancy and designed to investigate the origins of childhood conduct problems (for more information see: Sharp et al., 2012). The Wirral is a peninsula in the North West of England, UK, where socioeconomic conditions range from deprived inner city to affluent suburbs, but with low numbers from ethnic minorities. Sociodemographic characteristics are presented in online Supplementary Table S5. The whole cohort comprised 1233 mothers recruited during pregnancy; this analysis uses data collected from an intensive subsample of mothers and children at age 2.5 years (*n* = 253, mean age 2.58 years, s.d. = 0.19, 123 M 130F), and the whole cohort of mothers and children (*n* = 884; 425 M; 459F) at age 3.5 years (*N* = 827; mean age years 3.49, s.d. = 0.21; 396 M; 431F), 5 years (*N* = 770; mean age years = 4.89, s.d. = 0.31, 369 M; 401F), 7 years (*N* = 770; mean age = 7.36 years, s.d. = 0.33, 366 M; 404F), and 9.5 years (*N* = 744; mean age 9.48, s.d. = 0.43, 354 M; 390F). Within the full WCHADS general population cohort of mothers-to-be, a sub-sample stratified by psychological risk was drawn for more intensive investigation. This design enables intensive measurement to be employed efficiently with the stratified subsample, while weighting back to the whole cohort enables general population estimates to be derived. The stratification variable, mother's responses to a questionnaire at 20 weeks of pregnancy (recruitment) assessing psychological abuse in their current or recent partner relationship (Moffitt et al., 1997). The stratification variable was chosen for its known association with a variety of risk factors for early child development. All mothers scoring above the threshold for psychological abuse towards themselves or their partners at 20 weeks gestation were eligible for inclusion in the intensive sample plus a random selection from those below the threshold. Within the intensively assessed stratified sub-sample, 51% were drawn from women with high psychosocial risk and 49% from those with low psychosocial risk. Data at age 2.5 years were available on the intensive subsample only, data were collected from the whole sample at the other four time points. A weighting variable was therefore included at the age 2.5 time point to weight the estimates back to the full, population representative sample (see online Supplementary materials for more information). We used the ignorable missing data properties of all-available data maximum-(pseudo)likelihood estimators and survey-weights to adjust for stratification and potential selective attrition associated with background factors. Data are therefore assumed to be missing at random (missing based on observed variables). This allows drop-out to be selective, potentially related to any included covariates and any already observed outcome scores, for example drop-out at the 3rd assessment that was associated with problem behavior observed at the second assessment.

### Measures

#### Child behavior checklist CBCL (Achenbach and Rescorla, 2000, 2001)

Both studies used the CBCL at every assessment point, first the parent-report form for preschool children (CBCL 1½–5) then switching to the version for school-aged children and adolescents (CBCL 6–18) at the appropriate age. Evidence for the validity of both versions has been provided in children with autism (Pandolfi, Magyar, & Dill, 2009, 2012; Pandolfi, Magyar, & Norris, 2014). Items are rated on a 3-point Likert scale (0 = Never, 1 = Sometimes, 2 = Often) with rating periods of the last 2 (preschool form) or 6 (school age form) months and combined into syndrome scales. The current study used raw scores on the Anxious/Depressed, Aggressive Behavior and Attention Problems subscales to assess emotional, behavioral and attention problems. *T* scores were not used as these have a pronounced floor effect, and are thus not appropriate to use when examining developmental change.

### Predictors

#### Sociodemographic risk

Family income and maternal education were included as continuous scales (see online Supplementary Table S4 for descriptive statistics). In Pathways, mothers reported on their household income on a 12-point response scale from less than $5000 to more than $ 80 000 CDN. In WCHADS, mothers reported income in brackets from less than £ 10 000 to more than £ 70 000. In both samples, education was grouped as 1 = less than high school, 2 = high school, 3 = further education, 4 = undergraduate degree, 5 = postgraduate degree.

#### Autism diagnostic observation schedule (ADOS; Lord et al., 2000)

The ADOS is a semi-structured assessment administered by a trained clinician to assess autistic symptoms. Raw scores were generated for the ADOS domains of social-affect (SA) and repetitive and restrictive behaviors (RRB) and the calibrated severity score (CSS) derived for each domain was included in this analysis. The ADOS administered at time 1 was used for this analysis. See online Supplementary Table S3 for descriptive statistics.

#### IQ – Wechsler intelligence scales

The Wechsler Intelligence Scale for Children, fourth edition WISC-IV (Wechsler, 2012) FSIQ was used in Pathways at time 6 (8.5 years). This measure was unavailable for 185 children included in the analysis, both due to sample attrition and 62 children who completed the lab assessment but fell below the basal competence of the test. Children unable to reach the basal completed the Merrill-Palmer-R (MPR, Roid & Sampers, 2004) instead. The missing WISC scores were imputed using chained equations from the contemporaneously assessed MPR DQ but also from the parent reported Vineland Adaptive Behavior Scales (VABS, Sparrow, Cicchetti, & Balla, 2005) Communication standard score, and to reduce bias of estimated associations with the other covariates, the sex of the child, baseline autism SA and RRB CSS, family income (assumed ordinal), maternal education, site (complete) and the three CBCL scores (described fully in online Supplementary Materials S1). The chained equation approach also imputed any missing values of these other variables. Two hundred imputation replicates were generated to account for the imputed values being estimated rather than known. For the WCHADS children FSIQ at 9.5 years was from the two subtests form of the Wechsler Abbreviated Scales of Intelligence, second edition WASI-II (Wechsler, 2011). See online Supplementary Table S4 for descriptive statistics of FSIQ scores in both samples.

### Analysis plan

Individual latent curve models for aggression, anxious-depressed and attention problems skewed scores (over-dispersed Poisson) were fitted in Stata 17.0 gsem. Models are estimated in each sample and compared descriptively. To account for any effects of selective missingness of data we used a combination of maximum likelihood and multiple imputation for the Pathways sample and pseudolikelihood and weights for the WCHADS cohort (described fully in the online Supplementary Materials and Table S1).

Firstly, figures were generated which plotted the mean Pathways scores against percentiles from the WCHADS data. Plots were generated that show the fractional polynomial fit and confidence interval for the Pathways cohort, together with the 95th, 75th, and 50th (median) percentiles from the distribution of (scaled) scores from the WCHADS general population cohort. These were obtained by a lowess fit to centiles estimated at 10 ages using predictive mean matching to raw scores to account for non-normality. The comparison of unweighted and attrition weighted estimated profiles for the WCHADS cohort (see online Supplementary Materials Fig. S1) suggests these percentiles are likely conservative i.e. the Pathways children would be assigned a higher percentile had the percentiles been based on a population cohort with no attrition.

The fitted growth models allowed for a cubic spline for the mean age trend (though the cubic term was not required for anxiety-depression), random intercept and linear slope factors and a scaling factor to accommodate the change from the preschool (age 2–5) to school-age (age 6+) CBCL versions. The intercept factor (referred to as level) reflects the overall level of scores on each dimension and the slope factor (referred to as growth) reflects the change in score with age. Robust standard errors were used to allow for possible model mis-specification of the random elements of the model and any weighting, and estimates combined from the 200 multiply imputed datasets using the mi estimate and mi test commands.

The raw scores were plotted using a fractional polynomial fit and confidence intervals to compare growth profiles in the two cohorts. Raw scores were re-scaled for the estimated effects of change in CBCL version, and for IQ and sex in each sample to provide growth profiles for a reference child, a boy with an IQ of 90.

For Pathways we report risk ratio estimates and Wald-tests for the prediction of level and growth slope in the counts of behavioral symptoms by autism symptom severity, sociodemographic risk (maternal education and family income), FSIQ and sex. Recruitment site was included as a main effect on level (using the largest, Montreal, as reference category).

## Results

The descriptive statistics of Table 1 show the autistic cohort with consistently higher scores on all three CBCL dimensions compared to the TD cohort, and autistic girls received somewhat higher scores relative to autistic boys, whereas TD boys received higher scores relative to TD girls. Mean FSIQ was higher in the TD (104.53, s.d. = 18.68) compared to the autistic cohort (84.66, s.d. = 13.52) and after imputation to account for attrition and children unable to complete the basal, the estimated mean IQ was reduced to 77.38 (s.e. = 11.0).

**Table 1.** Mean raw scores for males, females and total sample for FSIQ and for each CBCL subscale at each age in the Pathways and WCHADS samples

	Autistic			TD		
Age	Boys	Girls	Total	Boys	Girls	Total
FSIQ						
8.5	84.52 (18.33), 41–130, *n* = 161	85.54 (21.05), 54–131, *n* = 26	84.66 (18.68), 41–131, *n* = 187			
9.5				105.83 (13.69), 66–143, *n* = 328	107.14 (13.36), 68–140, *n* = 363	106.52 (13.52), 66–143, *n* = 691
CBCL Anxious-depressed						
2.5	–	–	–	1.28 (1.65), *n* = 123	1.51 (1.75), *n* = 130	1.40 (1.7), *n* = 253
3.5	3.06 (2.59), *n* = 308	3.81 (2.98), *n* = 57	3.18 (2.67), *n* = 365	1.64 (1.78), *n* = 396	1.55 (1.63), *n* = 431	1.60 (1.7), *n* = 827
4	2.77 (2.5), *n* = 283	3.27 (2.86), *n* = 44	2.84 (2.55), *n* = 327	–	–	–
5	2.48 (2.56), *n* = 259	3.31 (2.74), *n* = 42	2.60 (2.6), *n* = 301	1.86 (2.13), *n* = 369	1.78 (1.96), *n* = 401	1.82 (2.04), *n* = 770
6.5	2.43 (2.35), *n* = 216	3.21 (2.88), *n* = 34	2.53 (2.44), *n* = 250	–	–	–
7.5	3.30 (3.03), *n* = 163	4.76 (3.48), *n* = 34	3.55 (3.16), *n* = 197	2.82 (2.85), *n* = 366	2.97 (3.08), *n* = 404	2.90 (2.97), *n* = 770
8.5	3.54 (3.39), *n* = 177	4.55 (4.63), *n* = 33	3.70 (3.62), *n* = 210	–	–	–
9.5	3.90 (3.48), *n* = 129	5.55 (4.76), *n* = 29	4.20 (3.79), *n* = 158	4.58 (3.35), *n* = 354	3.26 (3.55), *n* = 390	3.20 (3.45), *n* = 744
10.5	3.92 (5.49), *n* = 149	4.85 (4.05), *n* = 26	4.06 (5.3), *n* = 175	–	–	–
CBCL Attention problems						
2.5	–	–	–	2.46 (1.96), *n* = 123	2.16 (1.81), *n* = 130	2.30 (1.89), *n* = 253
3.5	4.55 (2.34), *n* = 308	4.53 (2.29), *n* = 57	4.55 (2.33), *n* = 365	2.32 (2.03), *n* = 396	1.68 (1.74), *n* = 431	1.99 (1.91), *n* = 827
4	4.26 (2.25), *n* = 283	4.34 (2.32), *n* = 44	4.27 (2.25), *n* = 327	–	–	–
5	4.05 (2.34), *n* = 259	4.24 (2.22), *n* = 42	4.08 (2.32), *n* = 301	2.09 (2.14), *n* = 369	1.5 (1.90), *n* = 401	1.79 (2.04), *n* = 770
6.5	4.05 (2.43), *n* = 216	3.65 (2.29), *n* = 34	3.99 (2.41), *n* = 250	–	–	–
7.5	8.64 (4.48), *n* = 163	8.68 (4.75), *n* = 34	8.65 (4.52), *n* = 197	3.83 (3.64), *n* = 366	2.51 (2.82), *n* = 404	3.14 (3.3), *n* = 770
8.5	7.54 (4.54), *n* = 177	7.94 (4.44), *n* = 33	7.6 (4.52), *n* = 210	–	–	–
9.5	8.43 (4.31), *n* = 129	8.86 (4.8), *n* = 29	8.51 (4.39), *n* = 158	3.77 (4.16), *n* = 354	2.44 (3.22), *n* = 390	3.07 (3.75), *n* = 744
10.5	7.22 (4.52), *n* = 149	7.27 (4.09), *n* = 26	7.23 (4.45), *n* = 175	–	–	–
CBCL Aggressive behavior						
2.5	–	–	–	8.57 (5.63), *n* = 123	7.45 (4.97), *n* = 130	8.00 (5.32), *n* = 253
3.5	13.32 (7.44), *n* = 308	13.44 (7.47), *n* = 57	13.34 (7.43), *n* = 365	8.26 (6.35), *n* = 396	6.82 (5.34), *n* = 431	7.51 (5.88), *n* = 827
4	12.17 (7.33), *n* = 283	12.23 (7.09), *n* = 44	12.17 (7.29), *n* = 327	–	–	–
5	11.03 (7.67), *n* = 259	12.55 (6.57), *n* = 42	11.25 (7.53), *n* = 301	7.38 (6.47), *n* = 369	5.87 (5.62), *n* = 401	6.60 (6.09), *n* = 770
6.5	10.13 (8.16), *n* = 216	11.74 (8.48), *n* = 34	10.35 (8.21), *n* = 250	–	–	–
7.5	6.62 (5.87), *n* = 163	9.24 (6.93), *n* = 34	7.07 (6.13), *n* = 197	4.58 (5.04), *n* = 366	3.44 (3.89), *n* = 404	3.98 (4.51), *n* = 770
8.5	6.09 (6.18), *n* = 177	7.36 (6.7), *n* = 33	6.29 (6.26), *n* = 210	–	–	–
9.5	6.17 (5.57), *n* = 129	9.45 (6.84), *n* = 29	6.77 (5.94), *n* = 158	4.28 (5.12), *n* = 354	3.31 (4.36), *n* = 390	3.77 (4.75), *n* = 744
10.5	5.48 (5.77), *n* = 149	7.12 (5.92), *n* = 26	5.72 (5.8), *n* = 175	–	–	–

*Note.* FSIQ, full scale IQ reported as: Mean standard score (Standard deviation), range; CBCL, Child Behavior Check List reported as: Mean raw score (Standard deviation), *n*.

### Percentile plots charting the differences between autistic and TD children

**Figure 1.**
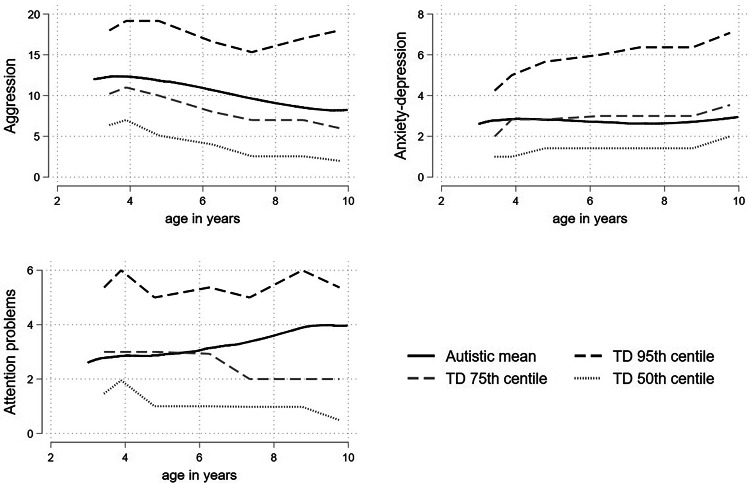
Fractional polynomial fit for the autistic cohort, together with the 95th 75th and 50th (median) percentiles from the distribution of (scaled) scores from the TD cohort for aggression, anxious-depressed, and attention problems.

**Figure 2.**
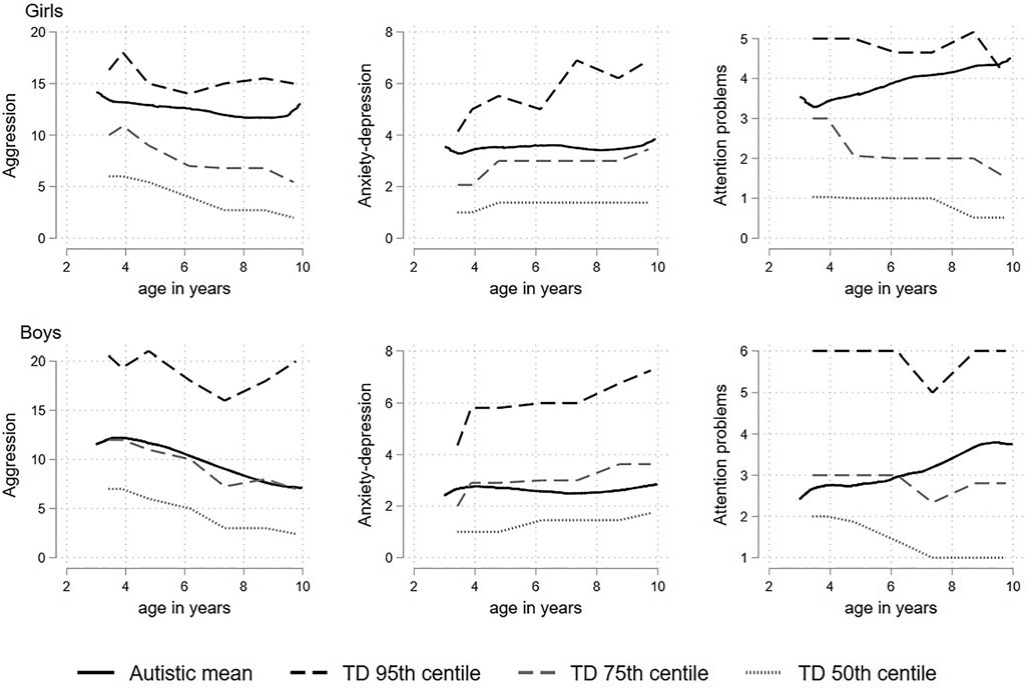
Fractional polynomial fit for the autistic cohort, together with the 95th 75th and 50th (median) percentiles from the distribution of (scaled) scores from the TD cohort for aggression, attention problems and anxious-depressed; girls shown in the top panel and boys shown in the bottom panel.

The percentile plots, which display the fractional polynomial fit for the autistic cohort mean, together with the 95th, 75th and 50th (median) percentiles from the distribution of (scaled) scores from the TD cohort are displayed in Fig. 1. The autistic children began around or above the 80th TD percentile for aggression and anxious-depressed and remained relatively stable around this point. For attention problems the autistic children began at the 75th TD percentile but by late childhood were closer to the 85th. In separate plots for boys and girls (Fig. 2) the autistic girls show strikingly higher mental health difficulties compared to the TD girls, with the mean aggression and anxious-depressed scores respectively tracking around the 90th and 80th centiles throughout childhood and attention problem scores beginning around the 80th centile and increasing to above the 95th centile by late childhood. Compared to same-sex peers, the autistic boys showed relatively fewer difficulties than the autistic girls, with scores tracking between the 65th and 75th centile and becoming more elevated in late childhood for attention problems.

#### Growth models examining predictors of level and growth in the autistic children

Table 2 shows growth model rate-ratio estimates examining whether income, maternal education, child sex, FSIQ and autism symptom severity in SA and RRB from the ADOS can explain firstly the variation in level of mental health problems in autistic children. Comparing intercept variance estimates for models with and without covariates, covariates explained 7% of the variation in level of aggression, 10% for anxiety-depression and 17% of hyperactivity. The strongest predictors were study site likely due to referral and recruitment differences. Income was negatively associated with aggression (RR = 0.87, *p* = 0.001), anxiety-depression (RR = 0.85, *p* = 0.004) and inattention (RR = 0.91, *p* = 0.003). Female sex was associated with the higher rates of anxiety-depression scores (RR = 1.23, *p* = 0.038). IQ was negatively associated with attention problems (RR = 0.91, *p* < 0.001) and ADOS Social-Affect symptoms were positively associated with attention problems (RR = 1.04, *p* = 0.005).

**Table 2.** Rate-ratio estimates from individual Poisson growth curve models with covariate effects on level and slope in the Pathways cohort the top half of the table displays the effects on level only and the bottom half displays the effects on level and slope<TE: Please check Table footnote.>

	Aggression		Anxious-depressed		Attention problems	
	RR (null = 1)	s.e.	RR (null = 1)	s.e.	RR (null = 1)	s.e.
Level only model						
Scale	1.42***	0.07	0.69***	0.07	0.46***	0.02
Spline age1	0.30***	0.10	0.72	0.10	0.75	0.21
Spline age2	1.83	2.92	1.05	2.92	1.02	1.49
Spline age3	0.27	0.76		0.76	0.62	1.61
Hamilton^2^	1.14	0.11	1.00	0.11	1.15	0.08
Halifax	1.43**	0.16	1.34*	0.16	1.29**	0.11
Vancouver	1.38***	0.12	1.05	0.12	1.26**	0.09
Edmonton	1.59***	0.16	1.47**	0.16	1.63***	0.13
WISC IQ	0.97	0.03	1.04	0.03	0.92***	0.02
Sex	1.00	0.08	1.23*	0.08	0.98	0.07
Mat. Educ.	0.98	0.03	0.99	0.03	0.99	0.03
Income	0.87***	0.04	0.84**	0.04	0.91**	0.03
ADOS SA css	0.98	0.02	0.99	0.02	1.04**	0.01
ADOS RBB css	0.97	0.02	1.00	0.02	0.99	0.02
Level variance	1.70***	0.14	2.26***	0.26	1.29***	0.07
Slope variance	11.35***	4.19	4.18***	1.10	1.83***	0.23
Lev-Slo covariance	0.46***	0.07	0.48***	0.07	0.78***	0.06
Level and slope model						
Scale	1.42***	0.07	0.69***	0.05	0.46***	0.02
Spline age1	0.05***	0.04	0.26	0.21	0.40	0.23
Spline age2	1.62	2.58	1.02	0.21	1.15	1.69
Spline age3	0.33	0.94			0.51	1.31
Effects on Level	1.15	0.11	1.00	0.11	1.15*	0.08
Hamilton	1.43**	0.16	1.33*	0.17	1.29**	0.11
Halfax	1.38***	0.12	1.04	0.11	1.26**	0.09
Vancouver	1.59***	0.16	1.47**	0.19	1.63***	0.13
Edmonton	1.01	0.04	0.99	0.05	0.97	0.03
WISC IQ	0.83	0.10	1.14	0.20	0.95	0.10
Sex	0.98	0.06	0.96	0.07	0.95	0.04
Mat. Educ.	0.89	0.06	0.72***	0.06	0.85**	0.05
Income	0.95	0.03	0.95	0.03	1.03	0.02
ADOS SA css	0.94	0.03	1.00	0.04	1.00	0.03
ADOS RBB css	1.42***	0.07	0.69***	0.05	0.46***	0.02
Effects on slope						
WISC IQ	0.89	0.06	1.11	0.08	0.91*	0.05
Sex	1.65*	0.41	1.15	0.33	1.06	0.18
Mat. Educ.	0.98	0.12	1.06	0.12	1.09	0.08
Income	0.94	0.12	1.34*	0.16	1.14	0.09
ADOS SA css	1.10	0.06	1.06	0.06	1.02	0.04
ADOS RBB css	1.09	0.06	1.00	0.05	0.98	0.04
Level variance	1.66***	0.13	2.17***	0.24	1.27***	0.07
Slope variance	9.68***	3.43	3.65***	0.94	1.75***	0.21
Lev-Slo covariance	0.48***	0.07	0.51***	0.08	0.80**	0.06
Observations	1971		1971		1971	
Participants	394		394		394	

*Notes*: ^1^Exponentiated coefficients, ^2^Montreal reference site, ^3^[variance estimates without covariates], ^4^*p < 0.05, **p < 0.01, ***p < 0.001.

In the extended models which included covariate effects on the slope, the covariates explained 6, 9 and 8% of the variation in the rates of change among children in aggression, anxiety-depression and attention problems respectively. For aggression, female sex was associated with significant relative decline (1.66, *p* = 0.047). Higher IQ was associated with decline in attention problems (RR 0.91, *p* = 0.047). Higher income was associated with higher relative increase in anxiety-depression (RR 1.35, *p* = 0.014) with weak evidence for it contributing to relative decline in attention problems (RR = 1.14, *p* = 0.116).

#### TD comparison adjusted for IQ and sex

**Figure 3.**
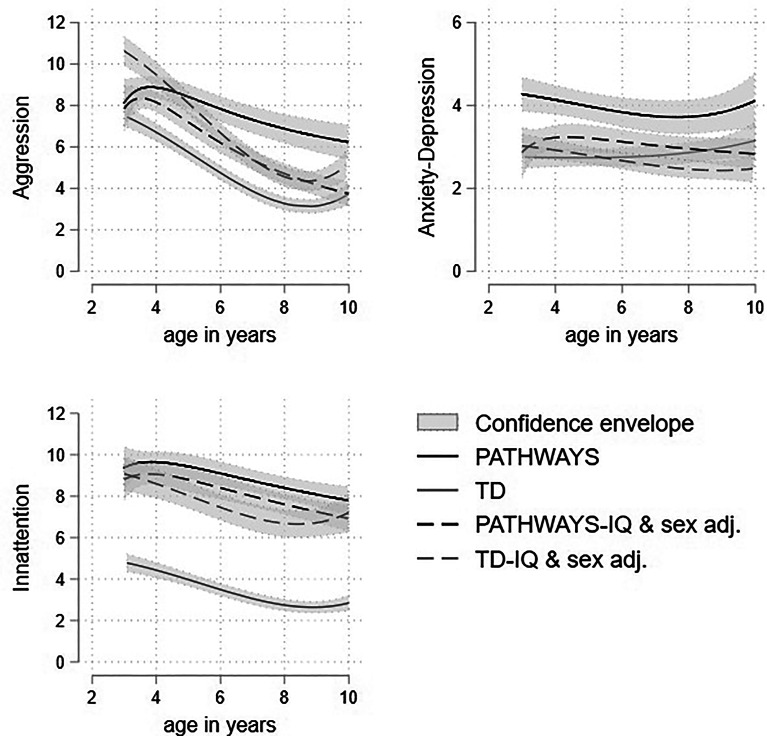
Trajectories of aggression, attention problems and anxious-depressed raw scores with 95% confidence intervals in the autistic and TD samples adjusted for male sex, preschool CBCL, and FSIQ = 90.

The Pathways and TD samples differed most strongly in IQ and proportion of boys. While the association of IQ and sex with mental health outcomes in Pathways was relatively modest, in TD samples they are more substantial (online Supplementary Table S6). Figure 3 shows the profile of scores for the Pathways and TD cohort both unadjusted, and adjusted to that expected for a boy with FSIQ of 90, a value 13 points higher than the Pathways mean and 15 points lower than the TD mean but lying well within the distribution of scores in both samples. Prior to adjustment for sex and FSIQ the profiles (represented as solid lines) are substantially elevated in the autistic cohort compared to the TD children for all three dimensions. However, the adjusted profiles fell much closer together, in the case of attention problems remarkably so. The autistic cohort scores are still elevated throughout most of development, though this difference is much smaller after accounting for sex and IQ differences. The growth patterns for all three dimensions in the two cohorts are largely similar, with the autistic cohort showing a less steep decline in scores over childhood.

## Discussion

This is the first study to compare mental health problems prospectively assessed from toddler/preschool age to late childhood in autistic and TD children. Using percentile plots and growth models we show higher levels of mental health problems in autistic compared to TD children, and small differences in growth patterns. The differences in level were substantially reduced by accounting for IQ and sex differences between the samples. Lower family income was associated with elevated level on all three dimensions, female sex was associated with higher anxiety-depression symptoms and both lower IQ and ADOS social-affect symptoms were associated with higher attention problems in autistic children.

Our findings are consistent with a large literature documenting increased mental health problems in autistic children (Salazar et al., 2015; Simonoff et al., 2008). We extend the literature in two ways, firstly by using a combination of growth curve modeling and percentile charts to show that mental health problems persist at a markedly elevated level from ages 2 to 10 years, tracking around the 75th to 85th centiles of TD children. Secondly, by showing that accounting for differences in sex and IQ between autistic and TD individuals substantially reduces the difference in level. Our results suggests that the higher overall rates of mental health problems in childhood in autism are largely explained by lower IQ and over-representation of males compared to the general population.

We observed very similar growth patterns between autistic and TD children, with a slightly steeper decline in symptoms from preschool age onward in autism, which may, in part, reflect regression to the mean due to behavioral problems contributing to referral and recruitment into the Pathways cohort. These findings differ from the two previous studies that compared autistic and TD children within large population cohorts which showed an increase in problems in autistic children (Colvert et al., 2021; Midouhas et al., 2013). However, these previous studies did not observe the decreases in behavioral or attention problems in the TD children which is typically reported from trajectory studies using the CBCL, and were found in the WCHADS sample. Consistent with studies of older autistic children (Anderson et al., 2011; Gotham et al., 2015), our results suggest that when using a more sensitive measure of mental health problems the growth patterns of autistic and TD children are more similar.

Whilst the evidence is very consistent for a decreasing trajectory of externalizing problems in TD children in early childhood, findings have been more mixed for internalizing problems. In Pathways, anxious-depressed problems were particularly elevated (~85th TD centile) relative to the TD children in the preschool period. In a recent study of young children (18–36 months) at elevated and typical familial likelihood for autism, elevated likelihood was associated with elevated anxious-depressed problems, over and above attention problems and aggression (Miller et al., 2019) suggesting that autism may be particularly linked to anxious-depressed problems in early childhood. Later in childhood, attention problems became more elevated in autistic relative to TD children in the current study. This is consistent with reviews noting that both the prevalence of ADHD in autistic children and the shared genetic influences increase with age (Leitner, 2014; Visser, Rommelse, Greven, & Buitelaar, 2016). It has also been hypothesized that the co-occurrence between ADHD and autism increases during adolescence as the demands on executive functions and social adaptation skills increase (Hartman, Geurts, Franke, Buitelaar, & Rommelse, 2016).

Consistent with the TD literature we found that lower family income was associated with elevated levels on all three dimensions. However, higher income was also associated with an *increase* in rates of anxiety-depression symptoms over childhood. This has not been reported previously, although the same direction of findings has been reported in relation to maternal education and level of emotional problems and neighborhood deprivation and decreasing growth in conduct problems in adolescence in Stringer *et al*.'s (2020) analysis of the SNAP cohort. In contrast, Gotham et al. (2015) found the expected association of lower maternal education and increasing growth in emotional problems from late adolescence to adulthood, but only in the group of children with limited vocabulary. In this study, we found no associations between maternal education and mental health dimensions in autistic children, only income was a significant predictor. Further research on the association between sociodemographic risk and mental health symptoms in autism is required.

The substantial reduction in differences in level between the TD and autistic sample after accounting for IQ reflected the strong associations between higher IQ and lower symptoms in TD. Within the autistic sample IQ was only significantly associated with attention problems, with higher IQ associated with lower level and with relative decreasing problems over childhood. Whilst the present study cannot test underlying mechanisms, studies with genetically informed designs have suggested shared genetic influences between IQ and ADHD problems (Kuntsi et al., 2004; Ronald, Simonoff, Kuntsi, Asherson, & Plomin, 2008). IQ may contribute to attention problems via frontally mediated deficits in executive functions (e.g. attention, planning, working memory and response inhibition; Sergeant, Geurts, & Oosterlaan, 2002; Van der Meere, Marzocchi, & De Meo, 2005), and externalizing problems may also interfere with children's cognitive development (Papachristou & Flouri, 2020).

The existing evidence on whether autism symptom severity predicts mental health problems is mixed. In their analysis of children diagnosed with autism within a general population cohort, Colvert et al. (2021) found overall ADOS severity was associated with level of hyperactivity. In the population-derived sample of autistic children Salazar et al. (2015) found positive associations with level of oppositional and some specific anxiety disorder symptoms. However, growth modeling studies of autistic children (Vaillancourt et al., 2017) and adolescents/adults (Stringer et al., 2020) have not found associations between autism symptom severity and mental health problems. Another publication on this sample using growth mixture modeling to identify trajectory groups found insistence on sameness assessed using the Autistic Diagnostic Interview was associated with belonging to high or increasing CBCL DSM anxiety symptoms classes compared to low symptoms (Baribeau et al., 2020). We examined severity in social affect and RRB separately, and found that social affect difficulties were significantly associated with elevated attention problems level only, consistent with Colvert et al. (2021). ADHD is the most commonly co-occurring disorder with autism and shares genetic heritability (Tick et al., 2016) and this has been shown to be greatest for the social communication difficulties element of the two disorders (Taylor, Charman, & Ronald, 2015). The association found in this study may reflect that shared heritability. In addition, the items used to assess attention problems, particularly in the school-age CBCL (e.g. ‘daydreams’, ‘stares blankly’) may overlap with social disconnectedness which may contribute to this association.

We found the pattern of sex differences in mental health problems among autistic children was not the same as that observed among TD children. Autistic girls showed higher levels of anxious-depressed problems, which is not observed until adolescence in TD. The lower rates of aggression and attention problems of TD girls was not seen, instead level was similar for autistic boys and girls, although there was a steeper decline over childhood in girls' aggression compared to boys'. The findings of no male-specific elevation in behavioral and attention problems is consistent with most studies of autistic children (Gadow et al., 2004; Prosperi et al., 2021; Salazar et al., 2015). Previous studies of sex differences in internalizing problems have been inconsistent. Our findings are consistent with one cross-sectional study of 3- to 18-year-olds which found elevated mother-rated but not teacher-rated emotional problems in females (Mandy et al., 2012). Building on recent approaches to characterizing the autism phenotype in females by characterizing difficulties relative to non-autistic females rather than autistic males (Lundström et al., 2019; Rødgaard et al., 2021) we showed that autistic girls experienced substantial elevations of mental health problems throughout childhood. Evidence suggests that females who receive an autism diagnosis show greater etiologic risk factors (e.g. a greater genetic mutational burden, Zhang et al., 2020) and that females who receive a diagnosis show higher levels of co-occurring intellectual disability or behavioral problems (Duvekot et al., 2017) compared to males. It may be that girls develop co-occurring conditions due to greater risk factors or that they need to experience a higher degree of difficulty to receive an autism diagnosis.

We examined associations between sociodemographic risk, IQ and autism symptom severity and mental health symptoms. Future research should examine a broader set of predictors known to be relevant to autistic children, for example, peer victimization (Rodriguez, Drastal, & Hartley, 2021), adverse life events (Carter Leno et al., 2022), social cognition (Carter Leno et al., 2019) and the potentially protective role of executive functioning (Johnson, 2012). In particular, research designs in which autistic girls are compared to non-autistic girls are needed to explain the substantially high rates of mental health problems experienced by autistic girls. In our autistic sample, the three mental health dimensions all followed a similar trajectory whereas there was a clearer divergence between the externalizing and internalizing growth trajectories in TD. Studies examining the general psychopathology or ‘*p* factor’ using the CBCL in TD samples over this age range have found that between 61–71% of variance in these three dimensions is explained by a shared general psychopathology factor. (McElroy, Belsky, Carragher, Fearon, & Patalay, 2018). The *p* factor has yet to be investigated in an autistic sample, it is possible that the greater similarity in the developmental trajectories found here in autistic children reflects even greater increase shared variance. Future research should also consider the role of autistic symptoms in general psychopathology (Ronald, 2019). Future studies should also use growth mixture modeling to identify possible groups of joint and individual trajectories of the three dimensions. Finally, it is important to note that the current sample focused on childhood with the oldest age around 10–11 years. In TD, early adolescence is a period of rapid increase in emotional symptoms in girls, whilst emotional symptoms decrease in boys and behavioral problems decrease in both boys and girls (Bongers, Koot, Van Der Ende, & Verhulst, 2004; Kwong et al., 2019). Future studies are needed which compare growth in mental health symptoms in autistic and TD adolescents to determine whether similar growth trajectories are still observed.

Strengths of this study include the longitudinal design with repeated assessment from early to late childhood. *Pathways* is an unusually large inception cohort of autistic children who were consecutively recruited from referrals at the participating clinical sites. WCHADS is a representative general population sample consecutively recruited at the sole provider of antenatal care in the region. We used the CBCL, which has been used in prior growth modeling studies of TD children, and is an in-depth measure with developmentally appropriate forms, and we accounted for the change in form in the analysis. Prior studies which have compared the course of mental health symptoms in TD and autistic children have not used measures which are suitable for both toddler/preschool and child age (Midouhas et al., 2013). Further, it seems likely that children within general population questionnaire-based studies who receive a diagnosis of autism have less representation of children with the full range of autistic symptoms than studies designed to recruit autistic children. *Pathways* and WCHADS were not designed to be used in a case–control analysis, but both studies used the CBCL repeatedly at similar age points. Both studies were conducted at similar points in time (2000's–2010's). However, it is a significant limitation that the studies are located in different countries and with different socio-demographic composition so we cannot discount the possibility that country-level differences contribute to the differences in level and growth between the two cohorts. There is, however, evidence that the CBCL operates similarly across culture, with a cross-country comparison of 45 different societies showing that only 10% of variance in parents' ratings is explained by culture or society (Rescorla, Althoff, Ivanova, & Achenbach, 2019). The *Pathways* sample is more affluent on average and more diverse than the WCHADS which may contribute to differences in mental health problems. Although we have controlled for deprivation in the analyses, the Wirral has higher levels of deprivation than the rest of the UK and deprivation is associated for increased risk for mental health problems (Rutherford, Sharp, Hill, Pickles, & Taylor-Robinson, 2019). Other differences include that the *Pathways* study sample is drawn from multiple sites across Canada whilst the WCHADS sample is drawn from one location in the UK. As the *Pathways* sample was recruited between ages 2 and 5 years, the findings may not generalize to individuals who are diagnosed with autism later in childhood, or in adolescence or adulthood. Related to this, studies of adolescents and adults that include self-report of diagnosis have a more even sex ratio than in the present study.

The small proportion of girls in the sample is also a limitation. This is characteristic of studies of autistic individuals diagnosed in early childhood, however, it does limit power to test for sex differences. Girls are typically diagnosed with autism later than boys (Rutherford et al., 2016) but are more likely to receive a diagnosis if they present co-occurring problems; the small proportion of girls in the *Pathways* sample may experience more co-morbidity than in studies with older children. Whilst the CBCL has been shown to be valid in autistic samples (Pandolfi et al., 2009, 2012, 2014) there is evidence that language ability may affect the level of symptoms endorsed, with children with lower language ability receiving lower internalizing scores than higher ability children (Fok & Bal, 2019). We imputed data for those children who were not able to reach the basal on the WISC to allow them to be included in the analysis, but due to small numbers we were not able to examine whether the results were different in children with low *v.* high cognitive ability. Finally, the measures used to assess FSIQ and collect sociodemographic information were similar, but not identical across studies. Different versions of the Weschler intelligence scales were used in the two studies at slightly different ages (8.5 years *v.* 9 years).

In conclusion, autistic children showed strikingly elevated levels of mental health problems throughout development compared to TD children, with similar growth patterns. However this difference in level was largely removed by accounting for IQ and sex differences between the autistic and TD sample. The predictors of mental health symptoms in autism showed some similarities to TD, but also some differences. Our findings suggest that assessment of mental health is an important component of care for children with autism, starting in early childhood. Clinicians may expect particularly elevated levels of anxious-depressed problems at preschool age and attention problems in later childhood. In particular, our findings highlight that girls who receive an early diagnosis of autism represent a group in high need of support for co-occurring mental health problems.

## Supporting information

Wright et al. supplementary material Wright et al. supplementary material

## Supplementary material

The supplementary material for this article can be found at https://doi.org/10.1017/S0033291723001599
