# Supplementary material for: A longitudinal comparison of emotional, behavioral and attention problems in autistic and typically developing children
Source: Psychol Med. 2023 Jun 29;53(16):7707–19. doi: 10.1017/S0033291723001599 (PMC10755241; doi:10.1017/S0033291723001599)
Supplement: Wright et al. supplementary material [file S0033291723001599sup001.docx]

**Supplementary Materials**

Supplementary Materials 1: Treatment of Missing Data

Pathways: For longitudinal data the generally preferred imputation strategy is to impute missing baseline/covariate data but to rely on the ignorable missing data properties of maximum likelihood for missing response data under the assumption of missing at random (Sterne et al., 2009). Imputation and analysis were undertaken using the Stata 17 mi impute, mi estimate and mi test suite of commands.

Missing values for phase 6 WISC standard scores, income (assumed ordinal), maternal education, and ADOS SA and RRB calibrated severity scores were imputed using the chained equations approach together with baseline, sex, site, CBCL aggression, anxious-depressed and inattention scores (assumed Poisson), and phase 6 Merrill-Palmer ratio DQ, and parent Vineland communication standard scores, the latter acting as auxiliary variables to provide more efficient imputation of the missing WISC scores and account for any gender, site and behavioural biases. The variables required for the potential time-varying effects of imputed variables were calculated as passive variables. Two hundred imputation replicates were generated. Table S1 below summarizes the imputed values with the last 2 variables being auxiliary only, and thus not included in the growth curve analyses. Of the cohort of 421 only 397 provided observed CBCL response scores enabling them to be included in the fitted growth curve models.

Table S1 Missingness among variables with incomplete data in the multiple-imputation model.

Variable | Complete Incomplete Imputed | Total

WISC FSIQ | 212 209 209 | 421

ADOS SA-CSS | 409 12 12 | 421

ADOS RRB-CSS | 409 12 12 | 421

Maternal Educ. | 377 44 44 | 421

Income | 375 46 46 | 421

VABSComm SS | 269 152 152 | 421

MP ratio DQ | 64 357 357 | 421

Two hundred imputed datasets were calculated, models estimated on each and results collated following Rubin’s rules (Rubin, 1987). The coefficients for plots over time of behaviour raw-scores and adjusted raw-behaviour scores were taken from the average estimates over the 200 imputations. The percentile plots were based entirely on the observed behavioural scores with no covariate adjustment.

The Stata imputation command used was:

mi register imputed iq VABS6cs MP6ra sacss rrbcss income mat_ed ag1 d1 p1

 mi impute chained (regress) iq VABS6cs MP6ra sacss rrbcss mat_ed ///

                  ag1 d1 p1 (ologit) income = sex dh dg dv de  ///

                  , add(200) force augment

WCHADS: Serving as a comparison sample of typically developing children, covariate adjustment was included only for the purposes of better comparison. Adjustments for sex and IQ seemed the most necessary but IQ was unavailable for some participants. In addition, while assessments 2 to 5 targeted the whole cohort, assessment 1 was stratified. Here we considered the most efficient approach was the adoption of sampling and attrition weights that included maternal age, smoking, education, exposure to psychological abuse, marital status and neighbourhood deprivation as predictors and the missing data properties of maximum pseudo-likelihood.

Growth Curve Models

Table S6 shows the parameter estimates for the full growth curve models with cubic spline for mean change, random intercept and linear slope, and all covariates on level and linear slope. Covariate effect estimates were very similar for unweighted and missing-data weighted models, with the mapping of significant covariate effects being identical for all three behavioural outcomes. Unadjusted and sex and IQ adjusted (adjusted to boys with IQ=90) behavioural fractional polynomial fitted age profiles are shown in Figure S1. For aggression and inattention, the weighted and unweighted profiles are very similar, with the weighted estimate profile falling a little lower than the weighted. For anxiety-depression covariate adjustment changed the shape of the profile, adjusted scores being lower for the older, with weighted estimates being slightly lower than unweighted before age 5 but increasing thereafter.

Estimation of WCHADS based percentile scores for Pathways children was based on unadjusted scores for boys and girls separately. Our comparison of unweighted and attrition weighted estimated profiles for the WCHADS cohort suggests these percentiles are likely conservative i.e. that Pathways children would have been assigned a higher percentile had the percentiles been based on a WCHADS cohort suffering no attrition.

References

Rubin, D.B. (1987) Multiple Imputation for Nonresponse in Surveys. John Wiley & Sons Inc., New York. doi.10.1002/9780470316696

Sterne, J. A. C., White, I. R., Carlin, J. B., Spratt, M., Royston, P., Kenward, M. G., Wood, A. M., & Carpenter, J. R. (2009). Multiple imputation for missing data in epidemiological and clinical research: Potential and pitfalls. In BMJ (Online) (Vol. 339, Issue 7713, pp. 157–160). British Medical Journal Publishing Group. doi.10.1136/bmj.b2393


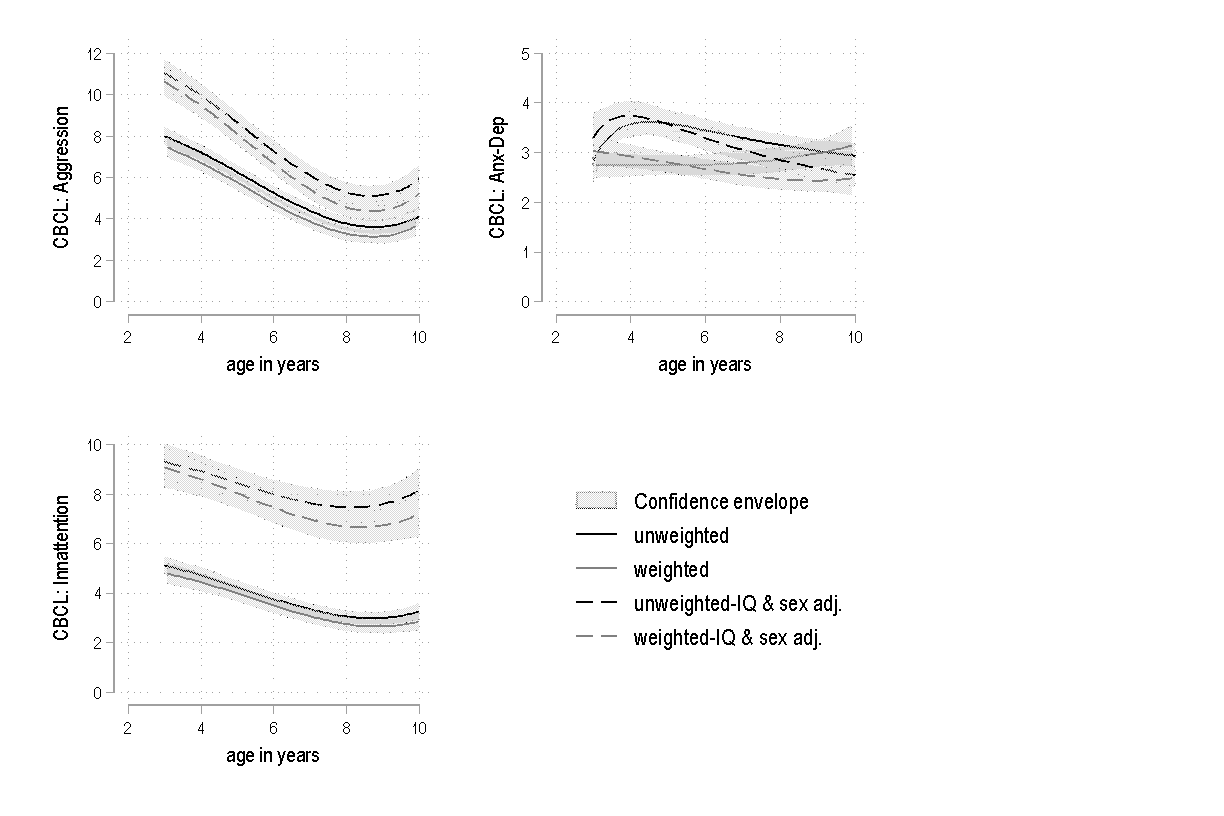


Figure S1 Fractional polynomial developmental profiles adjusted and unadjusted, weighted and unweighted for missing data.

Table S2: Site distribution in the Pathways cohort

| Site | N (%) |
| --- | --- |
| Montreal | 132 (33.2) |
| Vancouver | 90 (22.7%) |
| Hamilton | 60 (15.1%) |
| Halifax | 54 (13.6%) |
| Edmonton | 61 (15.4%) |

Table S3. ADOS module used at T1 in Pathways.

| Module | N (%) | | |
| --- | --- | --- | --- |
|  | Boys (n=334) | Girls (n=63) | Total (n=397) |
| 1 | 254 (76%) | 49 (77.8%) | 303 (76.3%) |
| 2 | 72 (21.6%) | 13 (20.6%) | 85 (21.4%) |
| 3 | 6 (1.8%) | 1 (1.6%) | 7 (1.8%) |
| Missing | 2 (0.6%) | - | 2 (0.5%) |

Table S4. Pathways participants diagnostic information

|  | Total Sample | Boys | Girls | *P** |
| --- | --- | --- | --- | --- |
| Age at diagnosis | 3.19 (.73), 1.60 – 4.96 | 3.19 (.73), 1.64 – 4.96 | 3.19 (.75), 2.00 – 4.83 | ns |
| ADOS SA CSS | 7.42 (1.85), 2 - 10 | 7.44 (1.86), 2 - 10 | 7.29 (1.81), 4 – 10 | ns |
| ADOS RRB CSS | 7.85 (1.71), 1 -10 | 7.89 (1.70), 1 - 10 | 7.63 (1.78), 1 - 10 | ns |
| ADOS total CSS | 7.59 (1.72), 2 - 10 | 7.63 (1.75), 2 - 10 | 7.40 (1.58), 4 – 10 | ns |

Note. Results presented as: Mean (Standard Deviation), Range. ADOS = Autism Diagnostic Observation Schedule. SA= Social Affect. CSS = Calibrated Severity Score. RRB = Restrictive and Repetitive Behaviors. WISC-IV *Difference tested using independent sample T-Test.

Table S5. Table 1: Sociodemographic characteristics of both samples

|  | Autistic (n=397) | | | TD (n=884) | |
| --- | --- | --- | --- | --- | --- |
|  | N | | % | N | % |
| Maternal education |  | | |  | |
| - Less than high school | 19 | | 5.1 | 10 | .1 |
| - High school completed | 34 | | 9.1 | 59 | 7.7 |
| - Further Education | 164 | | 41.9 | 429 | 56.1 |
| - Undergraduate degree | 111 | | 29.7 | 170 | 22.2 |
| - Postgraduate degree | 46 | | 12.3 | 98 | 12.8 |
| - Missing | 23 | | 5.1 | 1 | .1 |
| Ethnicity |  | | |  | |
| - White | 278 | 70.0 | | 862 | 97.5 |
| - Black | 14 | 3.5 | | 4 | .5 |
| - Asian | 47 | 11.9 | | 8 | .9 |
| - Mixed or other | 38 | 9.6 | | 10 | 1.1 |
| - Missing | 20 | 5.0 | | - | - |
| Family income^1^ |  | | |  | |
| - Less than 10 000 | 13 | | 3.3 | 60 | 6.8 |
| - 10 000 – 20 000 | 30 | | 7.5 | 94 | 10.6 |
| - 21 000 – 30 000 | 20 | | 5.0 | 117 | 13.2 |
| - 31 000 – 40 000 | 32 | | 8.1 | 149 | 16.9 |
| - 41 000 – 50 000 | 22 | | 5.5 | 127 | 14.4 |
| - 51 000 – 60 000 | 51 | | 12.8 | 109 | 12.3 |
| - 61 000 – 70 000 | 34 | | 8.6 | 49 | 5.5 |
| - More than 71 000 | 165 | | 41.6 | 60 | 6.8 |
| - Missing | 30 | | 7.6 | 119 | 13.5 |

1. Income is in CAD for the autistic sample and pounds for the TD sample

Table S6: Weighted and unweighted coefficients from individual Poisson growth curve models for the three mental health dimensions with covariate effects on level and slope

|  | Aggression | | | | Anxiety-depression | | | | Attention problems | | | |
| --- | --- | --- | --- | --- | --- | --- | --- | --- | --- | --- | --- | --- |
|  |  |  | Weighted | |  |  | Weighted | |  |  | Weighted | |
|  | b | se | b | se | b | se | b | Se | b | se | b | se |
|  |  |  |  |  |  |  |  |  |  |  |  |  |
| Scale version | 0.00 | 0.17 | -0.03 | 0.18 | -0.72^**^ | 0.26 | -0.62^*^ | 0.29 | -0.90^***^ | 0.21 | -0.96^***^ | 0.24 |
| Spline - age1 | 0.72 | 0.54 | 0.75 | 0.55 | 2.36^**^ | 0.74 | 2.11^**^ | 0.80 | 0.40 | 0.59 | 0.27 | 0.63 |
| age2 | -6.89^**^ | 2.61 | -7.52^**^ | 2.89 | -7.29 | 4.23 | -5.86 | 4.61 | -3.19 | 3.39 | -3.86 | 3.80 |
| age3 | 16.72^*^ | 6.52 | 18.41^*^ | 7.21 | 15.82 | 10.55 | 12.20 | 11.51 | 7.77 | 8.46 | 9.52 | 9.53 |
| Effects on level |  |  |  |  |  |  |  |  |  |  |  |  |
| IQ | -0.01 | 0.05 | 0.01 | 0.06 | -0.25^**^ | 0.08 | -0.27^**^ | 0.08 | -0.04 | 0.06 | -0.02 | 0.06 |
| Female Sex | -0.21^*^ | 0.09 | -0.24^*^ | 0.10 | 0.00 | 0.13 | 0.01 | 0.14 | -0.26^*^ | 0.11 | -0.29^*^ | 0.11 |
| Maternal Education | 0.10 | 0.06 | 0.06 | 0.06 | 0.12 | 0.08 | 0.09 | 0.09 | 0.03 | 0.07 | -0.02 | 0.08 |
| Income | -0.06 | 0.06 | -0.07 | 0.07 | -0.11 | 0.09 | -0.12 | 0.10 | -0.20^**^ | 0.07 | -0.22^**^ | 0.08 |
| Effects on slope |  |  |  |  |  |  |  |  |  |  |  |  |
| IQ | -0.23^*^ | 0.09 | -0.21^*^ | 0.09 | 0.27^**^ | 0.10 | 0.32^**^ | 0.12 | -0.32^**^ | 0.10 | -0.31^**^ | 0.10 |
| Sex | 0.13 | 0.16 | 0.15 | 0.17 | 0.10 | 0.18 | 0.12 | 0.19 | -0.19 | 0.17 | -0.16 | 0.17 |
| Mat. Educ. | -0.17 | 0.11 | -0.18 | 0.11 | -0.19 | 0.12 | -0.19 | 0.12 | -0.12 | 0.10 | -0.08 | 0.11 |
| Income | -0.16 | 0.11 | -0.14 | 0.11 | 0.10 | 0.12 | 0.13 | 0.13 | 0.09 | 0.11 | 0.08 | 0.12 |
| Constant | 1.80^***^ | 0.28 | 1.96^***^ | 0.29 | -0.00 | 0.37 | 0.01 | 0.38 | 2.01^***^ | 0.33 | 2.22^***^ | 0.34 |
| Intercept variance | 0.77^***^ | 0.10 | 0.79^***^ | 0.10 | 1.15^***^ | 0.15 | 1.12^***^ | 0.15 | 0.60^***^ | 0.11 | 0.62^***^ | 0.12 |
| Slope variance | 2.30^***^ | 0.26 | 2.23^***^ | 0.26 | 1.83^***^ | 0.26 | 1.81^***^ | 0.26 | 1.51^***^ | 0.26 | 1.38^***^ | 0.26 |
| Covariance | -0.73^***^ | 0.14 | -0.73^***^ | 0.14 | -1.04^***^ | 0.18 | -1.02^***^ | 0.18 | -0.39^**^ | 0.15 | -0.37^*^ | 0.16 |
| Observations | 2535 |  | 2535 |  | 2535 |  | 2535 |  | 2535 |  | 2535 |  |

Table S1 Unweighted and inverse probability weighted estimates of log-RR coefficients, standard errors and significance (p***<.001, p**<.01, p*<.05)
